# Locked nucleic acid oligomers as handles for single molecule manipulation

**DOI:** 10.1093/nar/gku760

**Published:** 2014-08-26

**Authors:** John P. Berezney, Omar A. Saleh

**Affiliations:** 1 Materials Department, University of California, Santa Barbara, Santa Barbara, CA 93106, USA; 2 Biomolecular Science and Engineering Program, University of California, Santa Barbara, Santa Barbara, CA 93106, USA

## Abstract

Single-molecule manipulation (SMM) techniques use applied force, and measured elastic response, to reveal microscopic physical parameters of individual biomolecules and details of biomolecular interactions. A major hurdle in the application of these techniques is the labeling method needed to immobilize biomolecules on solid supports. A simple, minimally-perturbative labeling strategy would significantly broaden the possible applications of SMM experiments, perhaps even allowing the study of native biomolecular structures. To accomplish this, we investigate the use of functionalized locked nucleic acid (LNA) oligomers as biomolecular handles that permit sequence-specific binding and immobilization of DNA. We find these probes form bonds with DNA with high specificity but with varied stability in response to the direction of applied mechanical force: when loaded in a shear orientation, the bound LNA oligomers were measured to be two orders of magnitude more stable than when loaded in a peeling, or unzipping, orientation. Our results show that LNA provides a simple, stable means to functionalize dsDNA for manipulation. We provide design rules that will facilitate their use in future experiments.

## INTRODUCTION

The field of single-molecule manipulation (SMM) involves the application of force to a single biomolecule, and the measurement of the biomolecule's static or dynamic mechanical response. SMM has advanced the understanding of various biophysical systems, impacting such areas as DNA mechanics, protein–DNA interactions and protein folding (1–4). SMM is a powerful technique because, as a single-molecule measurement, it eliminates the inherent averaging of bulk measurements and thus can reveal heterogeneous distributions within populations. Further, force itself is a fundamental thermodynamic parameter on par with temperature or solute concentration; thus control of force will perturb and probe the energetic landscape of a system. Finally, direct force measurements permit insights into biomolecular systems that exploit mechanics, revealing the key role played by force-dependent reactions in many areas of molecular biology. Thus, SMM is well positioned to explore multiple aspects of biomolecular systems.

All SMM techniques require the immobilization of the molecule of interest: the ends of molecule must be secured to a solid surface in order to apply force. Immobilization is often accomplished by adding functional moieties (e.g. antigens) to the two ends of the molecule of interest, which allows specific attachment to two functionalized (e.g. antibody-coated) surfaces. Thus, techniques used to add functional groups to typical biomolecules (e.g. DNA) represent an area of interest to the single-molecule biophysicist because they are a basic part of experimental design (5).

Typical DNA functionalization techniques add labeled nucleotides through polymerase chain reaction or other DNA polymerase reactions. These enzymatic techniques are widely used but limit experimental possibilities due to the extended protocols and low yields involved with producing more complicated DNA substrates. Some alternative approaches avoid such experimental hurdles (5). For example, nicking enzymes have been shown to be useful for SMM (6). Non-enzymatic DNA labeling techniques have also been developed; for example, one recent strategy employs peptide nucleic acid (PNA) oligomers with functional modifications. These oligomers add a functional group to a specific homopurine sequence in a DNA substrate through a simple DNA/oligomer binding step. This approach has been successfully used in single molecule experiments both to label DNA for fluorescence measurements as well as for SMM (7–9). However, their use requires incubation in a limited range of salt concentrations and purification steps involving elevated temperatures to reduce non-specifically bound oligomers. Nonetheless, the idea of a biomolecular handle which can be stably attached to a specific sequence within a long DNA molecule, using a simple mixing protocol, is very attractive for SMM experiments.

Locked nucleic acid (LNA) oligomers are a type of nucleic acid analog that show potential as biomolecular handles for SMM. These molecules are capable of forming sequence-specific triplex structures with homopurine dsDNA. This non-covalent binding allows functional groups to be easily added to non-terminal positions within long DNA molecules with one simple incubation step. LNA oligomers can spontaneously form LNA/DNA triplexes both specifically and stably in a wide range of buffer conditions (10). Because of their useful DNA-binding properties, LNA oligomers have been used in variety of ways to target dsDNA (11–13). Further, LNA oligomers retain the phosphate backbone, and thus high charge, of RNA and DNA; as a consequence, LNA is highly water-soluble and easier to handle than PNA, in which the uncharged peptide backbone leads to poor aqueous solubility (14). Additionally, mixed LNA/DNA oligomers (‘mixmers’) can be synthesized using phosphoramidite chemistry, adding another level of control to their design (14). However, the suitability of LNA for SMM has never been evaluated.

We describe the design and testing of LNA oligomers that act as biomolecular handles for mechanical measurements. Using magnetic tweezers, we measure the specificity of LNA binding to DNA substrates up to 48 kbp in length. We measure the stability of LNA triplexes by measuring the lifetime of LNA/DNA bonds under applied force. By applying force using different experimental geometries, we show that the stability of LNA handles against applied force is highly dependent on the geometry of pulling: the handles are much more stable in response to shear pulling than unzipping. Our results show that LNA oligomers bind DNA in a specific manner and with suitable stability for further SMM applications. We present some basic design principles for the use of LNA oligomers as biomolecular handles for DNA functionalization in SMM instruments.

## MATERIALS AND METHODS

LNA mixmers (Exiqon) were designed to form parallel triplexes with three target sequences: two within the lambda genome (4404–4418 and 39138–39155 bp) and one within the pMAL-p5x vector (4525–4537 bp). These target sequences were chosen because they represent unique sequences within the target substrate. The triplex forming oligomers alternate between DNA and LNA residues to maximize binding efficiency and stability (15) and carry a biotin moiety at the 3′ end which is connected by a flexible triethylene glycol linker. The sequences of the three LNA oligomers are as follows: LNA-1: 5′-Biotin-TEG-T^m^CT ^m^CCT C^m^CT ^m^CTT CTC -3′; LNA-2: 5′-TTT TCT TTT TT^m^C TTT TCT-TEG-Biotin-3′; LNA-3: 5′-Biotin-TEG-^m^CTT C^m^CC TCT TTC ^m^C-3′ (underline denotes LNA base, superscript m precedes methylated cytosine).

**Figure 1.**
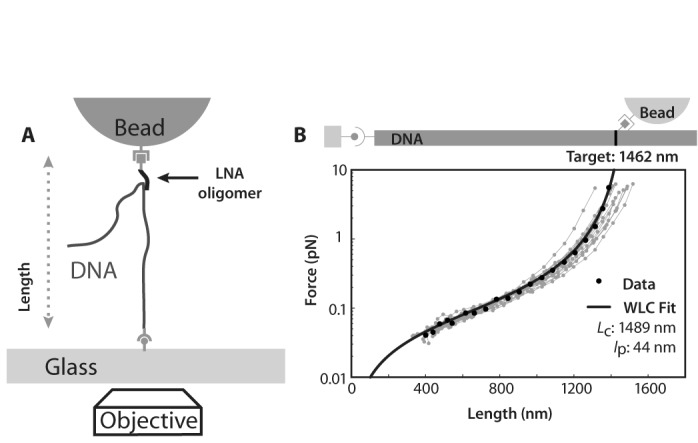
Overview of strategy to assay LNA/DNA binding strength and specificity in a magnetic tweezer: (**A**) Biotin-labeled LNA oligomers bind to surface-bound DNA, and mediate the tethering of a 1 μm streptavidin-coated magnetic bead. The tether is extended by a known force through application of a magnetic field, and its length is measured by optical bead tracking. (**B**) Force/length relations for individual tethers are fit to theory; one representative fit is plotted in black. The fits return estimates of each tether's persistence length, *l*_p_, and contour length, *L*_c_; the latter is an estimate of the LNA/DNA binding position. Multiplexed measurements permit elastic measurements of many tethers. Histograms of the fit contour lengths report on the characteristics of the NAA/DNA interaction. The 17 force/extension curves shown here correspond to the histogram (gray bars) in Figure 2A.

Target strands of DNA were prepared by adding a digoxigenin label to only one end of the dsDNA target. This modified end of the target substrate can then bind specifically to a surface while the other end remains free because it carries no functionalization. Once bound by biotin-labeled LNA oligomers, DNA molecules may be immobilized at the second end by streptavidin beads (Figure 1A). These tethered molecules can be measured using the magnetic tweezers because they are immobilized between the glass surface and the magnetic bead. However, the tethers are not rotationally constricted because there are multiple bonds which are free to rotate at the functional groups.

The lambda genome (48 502 bp) was functionalized using digoxigenin-labeled oligomers which were complementary to the cohesive sequences (IDT). The lambda genome was incubated with these *cos* labels and T4 DNA ligase 16 h at 4°C. Excess *cos* labels were removed using Amicon UItra 100 K spin columns. Two preparations of samples were produced: one with a digoxigenin label on the left terminal end and one with the label on the right terminal end. Thus, a specific terminal will bind to the surface while the other end is not functionalized.

The 5752 bp pMAL-p5x plasmid (NEB) was also prepared such that only one specific end was functionalized with digoxigenin. To linearize the plasmid and then de-activate the endonuclease, pMAL-p5x plasmid DNA (1 μg) was incubated at 37°C for 3 h with 10 units AvaI in 50 μl of 10 mM Tris–HCl, 10 mM MgCl_2_, 50 mM NaCl and then 20 min at 80°C. Then 100 nM nucleotides (dGTP, dATP, dCTP and dUTP-dig) were added with Klenow fragment (5 units) and incubated at 37°C for 5 h. The reaction was stopped by incubation at 75°C for 20 min. Due to the non-palindromic nature of the AvaI cut sites, only one overhang allows the incorporation of the dUTP nucleotides. Thus, the functionalized dUTP nucleotide was only incorporated at one end. The expected length of target-bound LNA-3 on the pMAL-p5x substrate is 1352 nm.

To probe the specificity of the LNA binding, the length of tethered DNA molecules in flow cells is measured with the magnetic tweezers. This method only measures DNA molecules to which a functional LNA mixmer has bound stably to the substrate DNA: molecules without the functional LNA cannot bind to the streptavidin beads used in the magnetic tweezers. Additionally, the specific labeling of the DNA substrates defines the orientation of the DNA within the magnetic tweezers. This allows the determination of the specific location of LNA binding on the DNA substrates by applying force to the tethers, measuring their extension, and calculating their contour length. By performing this multiple times, we can determine the frequency at which the LNA probes bind to sequences within the targeted DNA substrate.

Flow cells for SMM measurements were prepared by layering coverslips and double-sided tape to make a small chamber. The glass was cleaned using acetone, isopropyl alcohol and water washes, followed by plasma cleaner treatment. Finally, the slides were coated with Sigmacote. Typically, a flow cell was incubated 30 min with anti-digoxigenin to prepare the surface for DNA binding. This solution was washed out with 1x TE buffer (pH 7.5) and then incubated for 30 min with a BSA passivation buffer (50 mM MES, pH 7.0, 2 mM MgCl_2_, 20 mM NaCl and 0.2% BSA). The passivation buffer was washed out and a working buffer was flowed though (50 mM MES, pH 7.0, 2 mM MgCl_2_, 20 mM NaCl). Femtomolar concentrations of DNA reacted with LNA were flowed into the chamber and allowed to incubate for 2 h at 4°C. After incubation, the working buffer was used to rinse free LNA and DNA from the flow cell. Again, the passivation buffer was added to the flow cell and allowed to incubate for 10 min. One micron paramagnetic beads were added (myOne, Invitrogen), incubated for 10 min, and then a final rinse with working buffer removed unbound beads.

Magnetic tweezers were used to probe up to nine bead/DNA tethers at a time (16). The tweezers setup was used to apply forces to, and measure lengths of, tethered DNA molecules as described previously (17). Beads tethered by multiple DNA molecules were identified by anomalous height versus magnet rotation behavior and excluded (18).

LNA oligomers and digoxigenin labeled DNA were incubated in volumes of 10 μl for times between 10 min to 16 h in a reaction buffer (50 mM MES pH 7.0, 2–10 mM MgCl_2_ and 20–100 mM NaCl) at room temperature. The concentration of DNA was 5 nM and the LNA was 100× in excess of DNA. Once the reaction time was complete, the solution was diluted and used immediately for experiment. The concentrations of substrate DNA within the flow cells was controlled so that the yield of tethered beads was not be so large that separate DNA molecules could interact with the same magnetic beads, creating multiply tethered beads. The concentration of DNA added to the flow cell was diluted to 500 fM to eliminate multiply tethered beads while still maintaining a reasonable density of tethers.

## RESULTS

In this study we determined the stability and specificity of LNA oligomers bound to dsDNA to evaluate these nucleic acid analogs as sequence-specific biomolecular handles which can functionalize DNA for SMM instruments.

LNA/DNA hybridization was detected through the observation of tethered beads within flow cells (Figure 1A). In this experiment, magnetic tweezers measured the applied force and extension of DNA molecules tethered between two surfaces: a glass coverslip and a magnetic bead. DNA molecules were prepared with a single digoxigenin moiety on one end which can bind to anti-digoxigenin molecules attached to the surface of a glass coverslip. Simply incubating these DNA molecules in the flow cell leads to DNA immobilized on one end through this antibody interaction. The immobilization of the opposite end occurs via the LNA handles. LNA oligomers incubated with DNA form DNA/LNA triplexes(10). Because the LNA is synthesized with a biotin tag at one end, it is able to bind to streptavidin coated beads which are added into the flow chamber. Thus, the DNA bound by LNA is immobilized at one end through an antibody interaction and, at the other, through the biotin/streptavidin interaction via the DNA/LNA triplex. Only DNA incubated with LNA oligomers tethered the streptavidin-coated paramagnetic beads to the glass surface because streptavidin-coated magnetic beads can only tether to the surface through DNA with a biotin moiety such as the LNA oligomers; DNA not incubated with LNA formed no tethered beads.

Tetherable LNA/DNA constructs can be formed with simple protocols in short times. We found that LNA oligomers could be used to create tethered beads directly in a flow cell using LNA/DNA incubation times as short as 30 min. In addition to the detection of tethers within our single-molecule setup, we characterized LNA/DNA hybridization in bulk with the electrophoretic mobility shift assay (EMSA). The kinetics of the LNA binding within this experiment were fast enough that binding saturated within 1 h of incubation.

We used the magnetic tweezers to show that LNA oligomers bind with high specificity to the targeted DNA sequence. An important feature of our experiment is the ability to directly measure the position of bound oligomers, revealing the distribution of binding locations within a population of dsDNA:LNA complexes. Using the magnetic tweezers, we measured the relation between a molecule's extension and the applied force. We then fit this data to the Marko–Siggia wormlike chain force-extension model (19) and extracted estimates of the tether's contour length, *L*_c_, and persistence length, *l*_p_ (Figure 1B). *L*_c_ is the contour distance between the LNA/DNA bond and the surface-bound digoxigenin label, and thus gives the position at which the LNA binds to the DNA.

**Figure 2.**
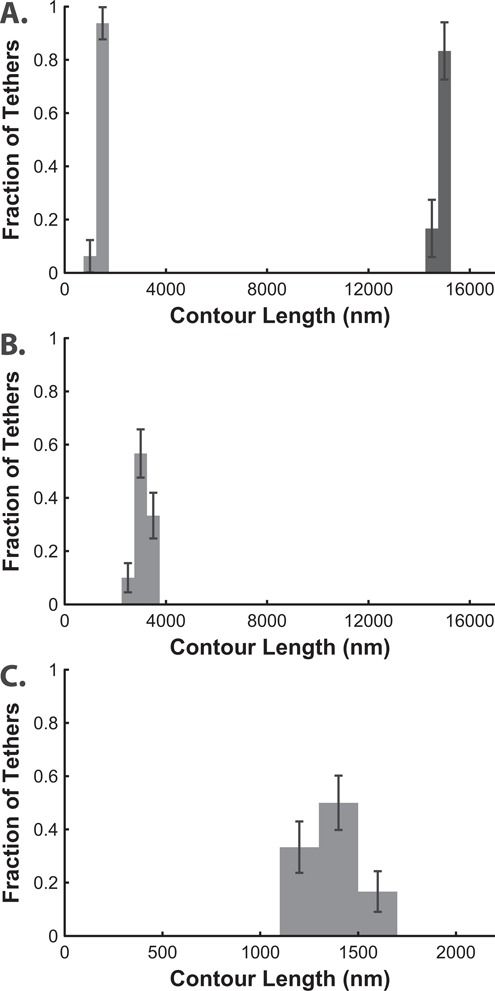
(**A**) LNA-1 oligomers bind specifically to the target position (1453 nm, 4404 bp) within the full 48.5 kilobases of the lambda phage genome, *N* = 17, bin = 500 nm (light gray bars). LNA oligomers also bind specifically when the lambda genome orientation is flipped (dark gray bars), *N* = 24. (**B**) LNA-2 oligomers bind specifically to the target position (39 138 bp, 3183 nm) within the lambda phage genome, *N* = 30, bin = 500 nm. (**C**) LNA-3 oligomers bind specifically to the target position (4525 bp, 1352 nm) within the pMAL-p5x vector, *N* = 24, bin = 200 nm. Error bars represent +/− 1 standard deviation.

We collected data on the binding position of a large number of LNA oligomers to a variety of substrates. Two LNA oligomers (LNA-1 and LNA-2) targeted two unique sites within the lambda genome and another LNA oligomer (LNA-3) targeted a unique site in the pMAL-p5x vector. Interrogating the whole lambda genome (48.5 kb) with the 15 nt LNA-1 oligomer resulted in a highly specific interaction (Figure 2A, light gray bars): no nonspecifically bound tethers were measured. When the orientation of the entire lambda substrate was flipped, a similarly high specificity was observed (Figure 2A, dark gray bars). The lambda genome was also interrogated with the 18 nt LNA-2 oligomer. Again, the histogram of binding locations shows highly specific binding around the targeted sequence (Figure 2B). Finally, a 13 nt handle, LNA-3, was used to functionalize the pMAL-p5x plasmid. The measured contour lengths of the tethers also were only of the expected length for specific binding to the target sequence (Figure 2C). Additionally, the mechanical properties of these molecules are in agreement with those of typical double-stranded DNA: in all experiments with DNA functionalized by LNA oligomers, measured mechanical behavior of the tethers resembled Marko–Siggia wormlike chain behavior with the average *l*_p_ = 42.5 ± 7.5 nm on the lambda substrate and 41.5 ± 9.0 nm on the pMAL-p5x substrate.

We found that LNA oligomers can withstand relatively high shear forces. Under piconewton-scale forces, weak noncovalent interactions can quickly break. We tested the mechanical stability of the LNA/DNA interaction by measuring the distribution of tether lifetimes at a constant applied force. Full length lambda DNA substrates bound by LNA were tethered to magnetic beads, and subjected to a constant force of 11.3 ± 0.3 pN; we then measured the time for each tether to cleave, as judged by the disappearance of the bead from the vicinity of the glass surface. The distribution of tether lifetimes displays an exponential decay with a 90 ± 7 min mean lifetime when using LNA handles, while tethers functionalized with enzymatically attached biotin and antidigoxigenin have a tether lifetime of 210 ± 9 min.

**Figure 3.**
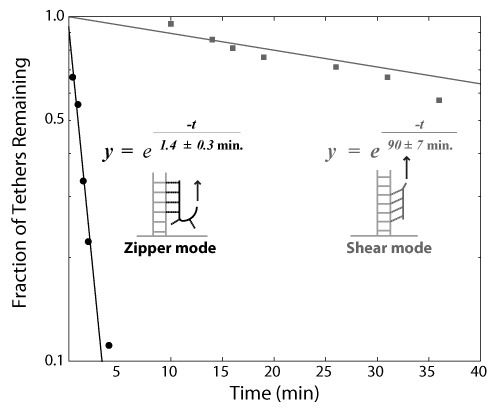
Stability of LNA oligomers in response to shear force (squares) is much greater than the stability in zipper mode (circles). Inset: When pulling in the shear direction, the applied force is distributed across the triplex bonds of the oligonucleotide. When pulling in the unzipping direction, the applied force sequentially destabilizes the bonds.

The stability of the triplex depends strongly upon the direction of applied force with respect to the bound LNA handle. We altered the orientation of the DNA substrate to alternatively apply force on the LNA/DNA triplex in a ‘zipper mode’ or ‘shear mode’ geometry. In the zipper mode, each Hoogsteen bond in the triplex is destabilized successively while, in shear mode, the applied force is distributed amongst the Hoogsteen bonds along the length of the LNA oligomer (Figure 3, inset). Similar single-molecule experiments on double stranded DNA have shown that Watson–Crick bonds are much more stable in the shear mode than the zipper mode (20,21). The number of surviving LNA/DNA tethers in the zipper geometry followed an exponential decay with a characteristic time of only 1.4 ± 0.3 min, approximately two orders of magnitude less than those which were tested in the shear geometry.

## DISCUSSION

The results show that DNA functionalization through hybridization of LNA oligomers leads to stable, specifically-bound handles appropriate for SMM via a simple incubation process. Using the magnetic tweezers, we showed DNA tethers composed with LNA handles withstand applied forces of 11.3 pN at time scales long enough to perform SMM experiments. These biomolecular handles are therefore an extremely simple tool to functionalize DNA in comparison with other methods typically used for SMM.

A variety of triplex forming nucleic acid analogs (NAAs) are available commercially but the two most widely used are PNA and LNA. Both of these DNA mimics boast high thermodynamic stability for triplex formation compared to the analogous DNA oligonucleotides (22,23). Additionally, these NAAs have better discrimination of mismatches than DNA triplex forming oligonucleotides. We experimented with PNA oligomers as well as psoralen modified LNA oligomers to investigate handles which might provide higher stability but found their binding to be highly non-specific compared to the results obtained with parallel triplex binding LNA oligomers (see Supplemental Material). We found that LNA has several advantages over other commercially available oligonucleotides. Firstly, it is produced with standard phosphoramidite chemistry and is, therefore, available at lower costs than PNA oligomers. Additionally, chimeras of DNA, RNA and LNA can be made, giving greater flexibility in oligomer design. Finally, unlike the protocols in which PNA is used to functionalize DNA, LNA handles do not require any post hybridization processing and the LNA/DNA reaction can be done directly in the flow cell before the experiment.

The LNA oligomers are much more stable when forces are applied in shear than in the zipper orientation. The measured values for tether lifetime differ by about two orders of magnitude. This result will be important for further design of LNA handles and has already been shown to be useful in similar applications (24). When force is applied in the shear orientation, the LNA handles have lifetimes that are about 57% shorter than DNA constructs labeled using enzymatic techniques, confirming our experience that LNA handles can be used to apply force to DNA molecules in SMM experiments. This stability is in agreement with previous results which show that LNA triplexes are stable in a variety of conditions (10). From the lifetime measurements, we calculate the lifetime of the 15 nt LNA triplex interaction to be 157 min at 11.3 pN. This time is well within the typical duration of SMM measurements and thus the LNA construct could easily be used in a wide array of SMM experiments.

Based on our experience, we provide a brief set of guidelines for using LNA oligomers as handles for SMM immobilization: First, we found that utilizing homopyrimidine sequences with alternating LNA and DNA nucleotides, and employing methylated cytosine were effective in creating successful hybridized constructs (10). Additionally, we found that triplexes 10 bases in length were not stable enough to withstand applied force; 13, 15 and 18 nucleotide oligomers were successfully used, thus we recommend using oligomers of at least 13 nt in length. The parallel triplex forming oligonucleotides studied in this paper target homopurine sequences. This limits the available targets. For example, in the lambda genome, there are ten homopurine sites with least 13 nt length (the minimum length we could mechanically probe) and only two >15 nt. Techniques such as molecular cloning could add specific target sites within substrates that do not have ideal sequences. Although LNA oligomers are able to discriminate between sequences with only a few mismatches, it is advantageous to pick target sequences with few one or two basepair mismatch sequences within the targeted DNA sequence. Finally, maximal stability results if the LNA triplexes are loaded in a shear, and not unzipping, geometry.

Immobilization of DNA via LNA handles does not require enzymatic steps; this is a major advantage over other immobilization techniques. Inherently, performing enzymatic steps to add functional moieties to DNA excludes the possibility of performing experiments on native biological structures because DNA must be purified before the beginning such protocols or because the enzymes themselves disrupt native DNA/protein structures. Additionally, incubation with LNA handles is less complicated than standard enzymatic reactions; it proceeds in a single step. These handles could allow the experimenter to apply single-molecule manipulation techniques to biomolecules excised directly from cells or even within cells. Technologically, we have shown that there is a drastic difference in stability between two mechanical geometries that could be an advantage in applications similar to those of single-molecule cut and paste which take advantage of differences in mechanical stability in various modes of pulling.

We have shown that LNA probes have the necessary stability to act as mechanical handles for SMM experiments, but that the stability of these handles varies strongly between different pulling directions. While LNA oligomers are robust against applied force in a shear geometry, when force is applied in the zipper mode, they are destabilized at relatively low forces. LNA oligomers also bind with high specificity to complementary sequences within very long dsDNA substrates. Since LNA/DNA binding is a simple, fast one-step process, use of NAA handles could lead to simpler experimental execution of current protocols.

## Supplementary Material

SUPPLEMENTARY DATA
