# Supplementary material for: Locked nucleic acid oligomers as handles for single molecule manipulation
Source: Nucleic Acids Res. 2014 Aug 26;42(19):e150. doi: 10.1093/nar/gku760 (PMC4231729; doi:10.1093/nar/gku760)
Supplement: SUPPLEMENTARY DATA [file supp_42_19_e150__index.html]

Locked nucleic acid oligomers as handles for single molecule manipulation — Locked nucleic acid oligomers as handles for single molecule manipulation — SUPPLEMENTARY DATA 

# Locked nucleic acid oligomers as handles for single molecule manipulation

## SUPPLEMENTARY DATA

**Files in this Data Supplement:**

- SUPPLEMENTARY DATA
