# Supplementary material for: Locked nucleic acid oligomers as handles for single molecule manipulation
Source: Nucleic Acids Res. 2014 Aug 26;42(19):e150. doi: 10.1093/nar/gku760 (PMC4231729; doi:10.1093/nar/gku760)
Supplement: SUPPLEMENTARY DATA [file supp_gku760_nar-03696-met-f-2013-File005.pdf]

Supplemental material for **Locked nucleic acid oligomers as handles for single molecule manipulation**

A variety of triplex forming nucleic acid analogs exist. The choice of oligonucleotide used as a handle can affect the stability of the handle bound to dsDNA. We measured the binding of two alternate designs to similar DNA constructs to evaluate them in reference to the parallel triplex binding LNA handles. In one case, we added a psoralen moiety to the end of an LNA oligomer; this change allows for the formation of covalent bonds with the targeted dsDNA sequence upon UV irradiation (26). In the other case, we used peptide nucleic acid (PNA) oligomers which could both form a triplex and invade the dsDNA, forming a highly stable “triplex invasion” bisPNA:DNA complex.

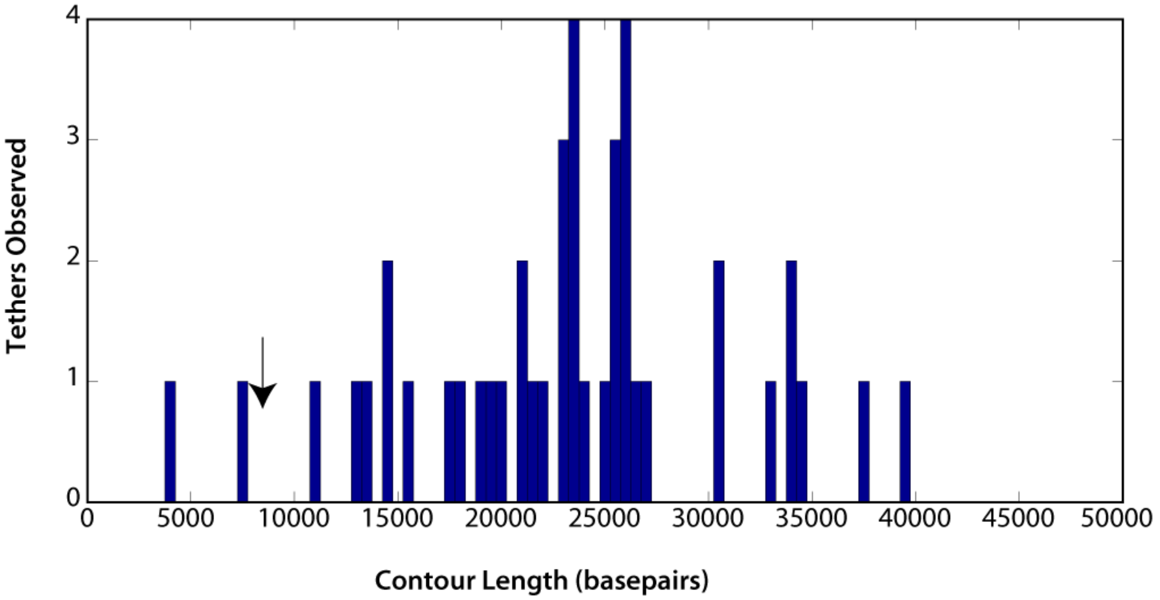

Supplemental Figure S1. Psoralen modified LNA handle showed extensive non-specific binding to the DNA substrate. This was attributed to the high affinity of psoralen to dsDNA. Target position was located at 8141 bp (arrow).

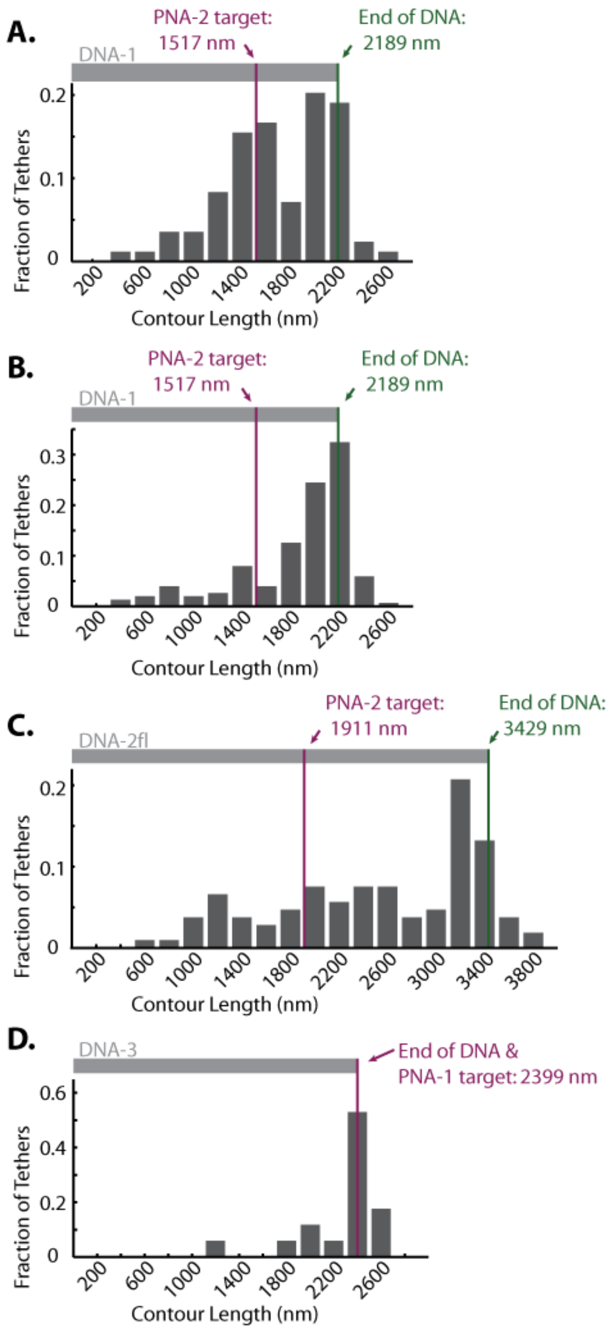

Supplemental Figure S2. Histograms of PNA binding locations indicate both target and extremity binding. (A) Two peaks occur – one at the position of the targeted sequence and one at the end of the DNA. *20 mM Na^+, 1 hour incubation, DNA-1, PNA-2. (N = 48)* (B) Increased [Na^+] increases the fraction of end-bound tethers. *110 mM Na^+, 1 hour incubation, DNA-1, PNA-2*. (*N = 151)* (C) The end-binding peak follows the position of the end of the substrate, independently of sequence. *20 mM Na^+, 10 minute incubation, DNA-2fl, PNA-2*. (*N = 54*) (D) Designing the PNA to target the end of the DNA molecule enables relatively specific binding at only one location. *110 mM Na^+, 1 hour incubation, DNA-3, PNA-1*. (*N = 17*).

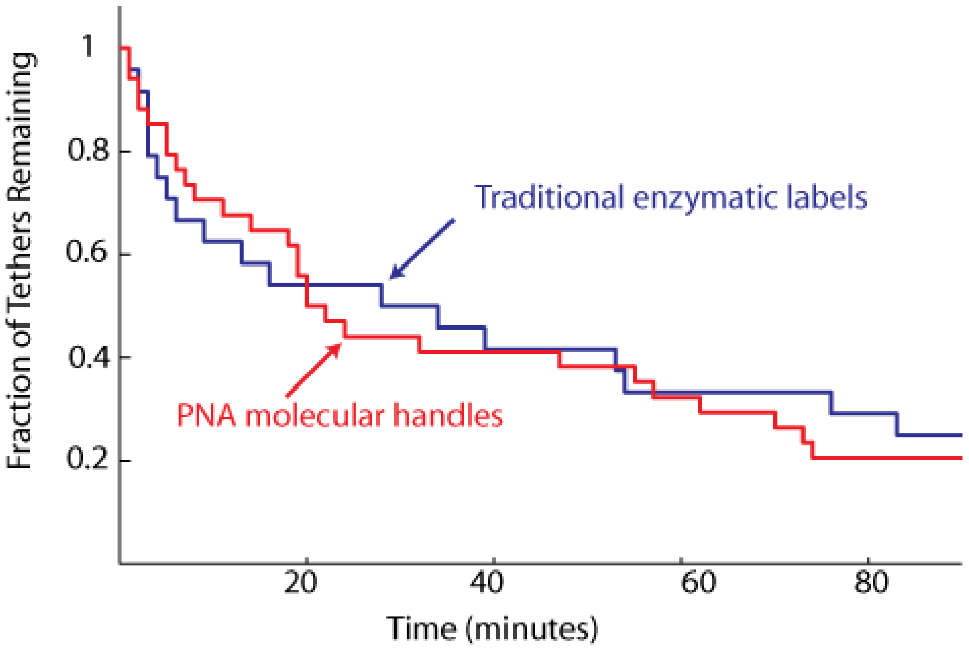

Supplemental Figure S3. PNA handles have similar mechanical stability to traditional methods. The lifetimes of enzymatically labeled DNA constructs (blue, 34 initial tethers) and PNA-hybridization labeled DNA constructs (red, 24 initial tethers) are statistically equivalent. Both constructs use the DNA-2 substrate; the PNA constructs were created with PNA-2 incubated for 1 hour with 110 mM Na^+.

Psoralen LNA handle

An 11 bp LNA oligomer with a 3' biotin moiety and an additional 5' modification of psoralen was designed to target an 11 bp sequence within the lambda genome. The oligomer (Exiqon) had the following sequence: 5' - /PsoC6/+TT+T/iMe-dC/+CT+TT+C/iMe-dC/+C/3bioTEG/ - 3'. This design was similar to that used before to target DNA where the psoralen moiety targeted a TpA position, allowing the formation of a covalent bond upon UV irradiation (12,26-28). We then measured the specificity using the same techniques as described in the full text. The results (Supplemental Figure S1) show that the binding was highly non-specific. Because of this non-specific binding, which we attributed to the stable non-specific binding to the binding of psoralen to TpA sites throughout the DNA, we did not find psoralen-modified handles to be useful for site-specific DNA functionalization.

PNA handle

Two bisPNA oligomers (Panagene) were designed to target separate 10 bp sequences in the lambda phage genome (GAGAGGGAAG, from 40,632 to 40,641 bp and AGGAAAGGA from 42,074 to 42,083 bp). The former oligomer, PNA-1, was designed with the sequence, Biotin – OOO – JTJ TJJ JTT J –OOO – CTT CCC TCT C – Lysine, while the latter oligonucleotide, PNA-2, was designed with the sequence, Biotin – OOO – TJJ TTT TJJ T – OOO – TCC TTT TCC T – Lysine ("O" is the flexible linker, 8-amino-3,6-dioxyoctanoic acid, "J" is pseudoisocytosine).

DNA for PNA targeting

For the PNA experiments, lengths of the lambda genome (NEB) were amplified using the Long Template PCR kit from Roche. One primer from each pair carried a digoxigenin label, such that the completed PCR products were digoxigenin-labeled on one end and unlabeled on the opposite end (primers from IDT). DNA-1 was 2,189 nm in contour length and extended from 37,585 to 44,059 bp in the lambda genome; DNA-2 was 5,449 nm and extended from 37,585 to 47,728 bp (thus containing the DNA-1 sequence). DNA-2fl was identical in sequence to DNA-2, but with digoxigenin at the opposite end. DNA-3 was 7,097 nm and extended from 40,632 to 47,709 bp.

PNA-DNA complex formation

PNA oligomers and digoxigenin labeled DNA were incubated in volumes of 10-50 µL at 37°C for various times (2 min to 1 week) in reaction buffer (1 mM EDTA, 10 mM sodium phosphate buffer (pH 7), 10 to 500 mM NaCl, and 10% acetonitrile). The concentration of DNA was between 1 and 5 nM and the PNA ranged from 1 to 200 times in excess of DNA. Once the reaction time was complete, the reaction was diluted 100x to 500 mM NaCl to stop the reaction and stored at 4°C.

Psol-LNA target DNA

The DNA used for psol-LNA:DNA hybridization was prepared in the same manner as the lambda DNA in the normal section of the paper. Simply, a *cos* sequence with a digoxigenin label was ligated onto one end of the lambda genome DNA.

PNA results

PNA/DNA binding was tested with four DNA substrates: three in which the PNA targeted an internal sequence (DNA-1, DNA-2, DNA-2fl), and one in which an extremity sequence was targeted (DNA-3). In all cases, substrates incubated with PNA oligomers form a significant number of tetherable constructs. Supplemental Figure S2 shows histograms of compiled contour lengths from several experiments. These plots show extensive non-specific binding, particularly at the DNA extremities. This non-specific end-binding moves with the end of the DNA as the DNA substrate is changed.

Psol-LNA results

Psol-LNA oligomers were hybridized with lambda DNA. Tests of similar LNA designs without psoralen modifications suggest that LNA oligomers of 11 bp are very unstable in response to force and the application of 3-5 pN is highly destabilizing. Nonetheless, the psoralen modified LNA oligomers

formed stable handles on the dsDNA and we used the magnetic tweezers to collect force-extension data, extracted the contour lengths from WLC fits and compiled histograms of tether binding positions. These histograms of binding sites show that the psol-LNA oligomers bound to the DNA non-specifically (Supp. Figure S1). There were no positions within the DNA at which the LNA oligomers seemed to bind preferentially, indicating non-specific binding to the dsDNA substrate.

Analysis of the mismatch sites within the substrate DNA could not explain the observed non-specific binding. We attribute the stable non-specific binding to the binding of psoralen to TpA sites throughout the DNA, forming covalently bound complexes at these sites.

PNA Tether lifetime

Using techniques described in the full text, we studied the lifetime of tethers composed with PNA oligomers. We prepared two sets of substrates, one with a biotin label carried by PNA and the other with a biotin label covalently attached by standard enzymatic methods. Both substrates were tethered to magnetic beads, and subjected to a constant force. Distributions of tether lifetimes under 13 pN for both substrates display a roughly exponential decay with a 40-50 minute characteristic time (Supp. Figure S3). Indeed, with 99% certainty, the two populations are statistically identical, as determined by a Kolmogorov-Smirnov test; this indicates both sets of tethers share the same weakest link (likely the dig/anti-dig bond), and thus that the PNA/DNA bond is not limiting.
